# Raf激酶抑制蛋白在非小细胞肺癌中的表达及其临床意义

**DOI:** 10.3779/j.issn.1009-3419.2012.10.06

**Published:** 2012-10-20

**Authors:** 大运 杨, 战 齐

**Affiliations:** 1 050011 石家庄，河北医科大学第四医院内科 Department of Internal Medicine, the Fourth Hospital of Hebei Medical University, Shijiazhuang 050011, China; 2 050011 石家庄，河北医科大学第四医院胸外科 Department of Thoracic Surgery, the Fourth Hospital of Hebei Medical University, Shijiazhuang 050011, China

**Keywords:** 肺肿瘤, Raf激酶抑制蛋白, Western blot, RT-PCR, 免疫组织化学, Lung neoplasms, Raf kinase inhibitory protein, Reverse transcription-polymerase chain reaction, Western blot, Immunohistochemistry

## Abstract

**背景与目的:**

Raf激酶抑制蛋白（Raf kinase inhibitor protein, RKIP）属于磷脂酰乙醇胺结合蛋白家族的成员，RKIP参与ERK/MAPK、G蛋白偶联受体和NF-κΒ等信号传导过程，且RKIP的表达减弱或丢失与多种肿瘤的发生发展及侵润转移相关。本研究旨在探讨RKIP在非小细胞肺癌（non-small cell lung cancer, NSCLC）组织中的表达及其与NSCLC临床病理特征的相关性。

**方法:**

应用RT-PCR、Western blot及免疫组化方法检测83例NSCLC及其癌旁组织标本中RKIP的表达，并结合临床病理学资料进行统计学分析，所有病例均经病理诊断确诊，均无其它部位原发肿瘤，术前无化疗、放疗和免疫治疗史。

**结果:**

NSCLC中RKIP mRNA及蛋白的表达明显低于癌旁组织，差异有统计学意义（*P* < 0.05）。RKIP与肿瘤分化程度、TNM分期、有无淋巴结转移及生存期有关（*P* < 0.05），但与患者的性别、吸烟、年龄及肿瘤的大小无关（*P* > 0.05）。

**结论:**

RKIP的低表达与NSCLC的发生及侵袭转移有关，可作为NSCLC预测及预后评估的指标。

## Abstract (en)

**Background and objective:**

Raf kinase inhibitory protein (RKIP) belongs to the phosphatidylethanolamine binding protein family. RKIP is an endogenous inhibitor of the ERK/MAPK, NF-κΒ, and G protein-coupled receptor signaling pathways. This study aims to investigate the expression of RKIP in non-small cell lung cancer (NSCLC) and to determine the association of RKIP expression with the clinicopathologic features of NSCLC.

**Methods:**

Reverse transcription-polymerase chain reaction, Western blot, and immunohistochemistry were used to detect RKIP expression in 83 specimens with NSCLC and normal lung tissues and to analyze the association of RKIP expression with the clinicopathologic features of NSCLC. All cases were confirmed by pathological diagnosis, and primary tumors were not found at other sites. No medical records of preoperative radiotherapy, chemotherapy, and immunotherapy were found in the groups.

**Results:**

RKIP expression was down-regulated significantly in NSCLC compared with adjacent cancer tissues (*P* < 0.05). It was associated with differentiation, pathological tumor-node-metastasis stage, survival time, and lympho invasion (*P* < 0.05) but not with gender, smoking status, age, tumor size, and histologic type (*P* > 0.05).

**Conclusion:**

The deficiency of RKIP expression is positively correlated with carcinogenesis and invasion metastasis of NSCLC. RKIP is a potential marker and target for clinic therapy.

Raf激酶抑制蛋白（Raf kinase inhibitor protein, RKIP）属于高度保守的磷脂酰乙醇胺结合蛋白（phosphatidylethanolamine binding protein, PEBP）家族的成员，是近年来发现的一种新的肿瘤转移抑制因子，其相对分子量约为21, 000-23, 000，定位于人类染色体12q24.23，其mRNA长1, 507 bp。研究^[1, 2]^发现，RKIP参与对ERK/MAPK、G蛋白偶联受体和NF-κΒ信号通路的调控，在细胞生长、增殖、分化、凋亡等生理过程中发挥重要作用，且RKIP与肿瘤转移密切相关。本研究采用RT-PCR、Western blot及免疫组化方法检测肺癌组织及其癌旁正常组织中RKIP的表达，以探讨RKIP与肺癌发生发展的关系。

## 1 材料与方法

### 1.1 一般资料

收集河北医科大学第四附属医院胸外科2009年6月-2011年6月83例肺癌患者手术切除的肺癌组织和正常肺组织。癌组织取自肿瘤中心处，正常肺组织取自距肿瘤边缘 > 5.0 cm处。其中男性48例，女性35例；年龄33岁-75岁，平均年龄53.7岁； < 50岁者43例，≥50岁者40例；按照WHO（2004）组织病理学分类标准分为：鳞癌32例，腺癌40例，腺鳞癌8例，大细胞癌3例；按细胞分化程度较好、居中、较差分为高分化25例，中分化23例，低分化35例；肿瘤长径D≥3.0 cm患者41例， < 3.0 cm患者42例；有淋巴结转移43例，无淋巴结转移40例；Ⅰ期患者18例，Ⅱ期患者20例，Ⅲ期患者29例，Ⅳ期患者16例。所有病例均经病理诊断明确，术前未行放、化疗和免疫治疗史。标本取材后部分置于液氮中速冻，然后转至-80 ℃冰箱中保存；部分标本用10%甲醛固定，石蜡包埋，作免疫组化染色。

### 1.2 试剂

Trizol试剂（上海invitrogen公司），RT-PCR试剂盒（美国Promega公司），TBST（10 mmol/L Tris-HCl, pH7.5, 150 mmol/L NaCl, 0.1%Tween-20），RKIP一抗为兔抗人多克隆抗体，二抗为辣根过氧化物酶（Horseradish peroxidase, HRP）标记的山羊抗兔多克隆抗体，内参磷酸甘油醛脱氢（glyceraldehyde-3-phosphate dehydrogenase, GAPDH）为单克隆小鼠抗人GAPDH抗体，二抗为HRP标记的山羊抗小鼠多克隆抗体（均为Santa Cruz公司，美国），ECL发光液（Pierce公司，美国），BCA蛋白浓度测定试剂盒为国产碧云天产品。即用型兔抗人RKIP多克隆抗体购自美国Santa Cruz公司。免疫组化S-P试剂盒和DAB显色试剂均购自北京中杉金桥生物技术有限公司。

### 1.3 实验方法

#### 1.3.1 RT-PCR法

##### 1.3.1.1 总RNA提取

按照说明书采用Trizol一步法抽提肺癌及癌旁正常肺组织总RNA，所得RNA溶于焦碳酸二乙酯（DEPC）水中。琼脂糖凝胶电泳验证RNA完整性，紫外分光光度计检验纯度并定量，所得RNA的吸光度（A_260_/A_280_）均在1.8-2.0范围内。

##### 1.3.1.2 RT-PCR检测RKIP mRNA表达

RKIP上游引物5’-GAATAGACCCACCAGCAT-3’，下游引物5’-CGTAAACCAGCCAGACAT-3’，扩增片段长度为236 bp。内参GAPDH的上游引物5’-ATCTGGCACCACACCTTCTACAATGAGCTGCG-3’，下游引物：5’-CGTCATACTCCTGCTTGCTGATCCACATCTGC-3’，扩增片段长度为342 bp。扩增条件为：95 ℃ 3 min，94 ℃ 45 s，53 ℃ 45 s，72 ℃ 1 min，35个循环；72 ℃ 5 min。取PCR产物5 μL产物加1 μL溴酚蓝，经1.5%琼脂糖凝胶电泳检测。电泳结果用DNA凝胶成像扫描仪（美国Fotodyne公司）摄像，Gel-Pro Analyzer 3.1软件测定条带吸光度，以（条带灰度×面积）/（参照物的灰度×面积）作为RKIP mRNA表达水平的参数，对RKIP产物相对定量。

#### 1.3.2 Western blot检测RKIP蛋白的表达变化

将液氮中冻存的组织标本取出称重，按每200 mg组织加1, 000 μL蛋白裂解液，置匀浆器中反复研磨，使组织充分匀浆化。冰浴60 min后，低温离心12, 000 r/min离心15 min，取上清，用BCA法测上清蛋白浓度，取100 μg蛋白进行聚丙烯酰胺凝胶电泳，聚偏氟乙烯膜电转移，含5%脱脂奶粉的TBST溶液封闭非特异性抗原，然后分别加入RKIP兔抗人多克隆抗体（1:1, 000）及GAPDH小鼠抗人单克隆抗体（1:1, 000），4 ℃孵育过夜，次日用TBST液洗膜3次，每次10 min，加入HRP标记的山羊抗兔IgG（1:2, 000）和HRP标记的山羊抗小鼠多克隆抗体（1:2, 000）37 ℃孵育1 h，TBST洗膜3次，每次10 min，ECL化学发光试剂自显影。RKIP相对含量用RKIP/GAPDH灰度比值表示，灰度采用Quantity One软件（Bio-Rad公司，美国）分析。

#### 1.3.3 免疫组织化学染色法

对石蜡切片进行免疫组化链霉素抗生物素蛋白-过氧化物酶（streptavidin-perioxidase, S-P）法测定。免疫组织化学染色程序按试剂盒说明书进行，主要包括石蜡切片、常规脱蜡至水，抗原修复、3%过氧化氢阻断内源性过氧化物酶、山羊血清封闭、滴加一抗、二抗及链霉素抗生物素蛋白-过氧化物酶复合物、DAB显色、对比染色封片。以已知正常乳腺组织阳性切片作阳性对照，以PBS代替一抗作为阴性对照。结果判定RKIP以胞质或胞膜出现棕黄色颗粒视为阳性。采用二次计分法：每例标本随机计数5个高倍视野（×400），计数每个高倍视野中阳性细胞所占百分比并计分，0分，无着色；1分，≤10%；2分，11%-50%；3分，51%-75%；4分，≥75%。肿瘤细胞按染色强度分为4等：0分为无染色；1分为浅黄色；2分为棕黄色；3分为棕褐色。用染色强度计分和染色百分比计分的乘积作为判断表达结果，若积分≤1为阴性， > 1为阳性。

### 1.4 统计学分析

应用SPSS 13.0软件进行统计分析。采用*t*检验、*F*检验和χ^2^检验进行数据分析。*P* < 0.05为差异有统计学意义。

## 2 结果

### 2.1 RKIP的RT-PCR检测结果

在NSCLC中，肺癌组织中RKIP mRNA的表达水平（1.213±0.325）较正常组织（1.963±0.232）低，两组相比差异有统计学意义（*t*=3.253, *P* < 0.05）（图 1）。

**1:**
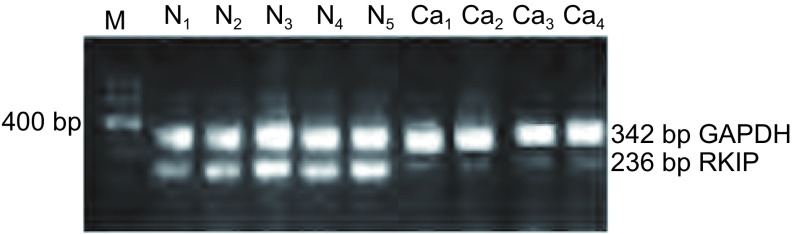
不同肺组织中RKIP mRNA表达的RT-PCR结果。M：分子量标志；N：正常肺组织；Ca：肺癌组织 Expression of RKIP mRNA in different lung tissues were detected by RT-PCR. M: marker; N: normal lung tissue; Ca: lung cancer tissue

### 2.2 RKIP的Western blot检测结果

NSCLC中RKIP蛋白在肺癌组织中的表达水平（0.629±0.224）较正常肺组织低（1.183±0.207），两组相比差异有统计学意义（*t*=3.146, *P* < 0.05）（图 2）。

**2:**
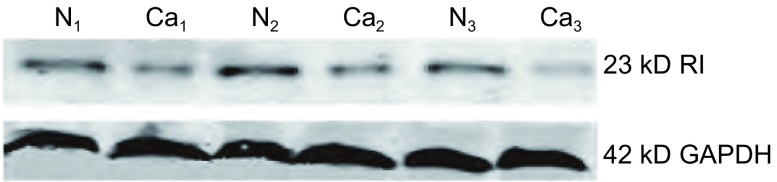
不同肺组织中RKIP蛋白表达的Western blot结果。N：正常肺组织；Ca：肺癌组织 Expression of RKIP protein in different lung tissues were detected by Western blot. N: normal lung tissue; Ca: lung cancer tissue

### 2.3 RKIP的免疫组化染色结果

 RKIP的阳性染色产物主要位在胞质或胞膜上。肺癌组织中RKIP的表达低于癌旁正常肺组织，RKIP在肺癌组和正常组表达的阳性率分别为42.2%（35/83）和79.5%（66/83），两组相比差异有统计学意义（χ^2^=24.299, *P* < 0.05）（图 3）。

**3:**
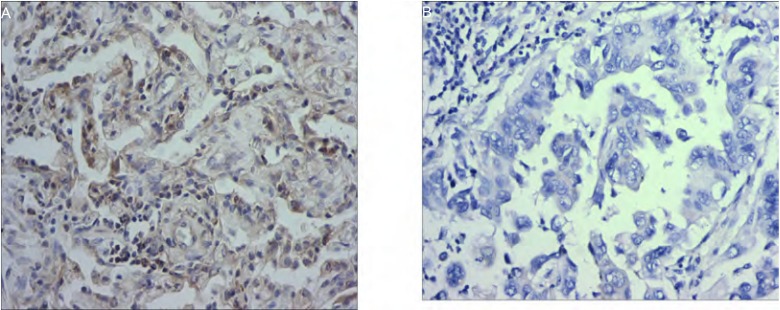
RKIP在肺腺癌中免疫组化染色（SP, ×200）。A：RKIP+；B：RKIP- Immunohistochemical staining of RKIP in lung adenocarcinoma (SP, ×200). A: RKIP+; B: RKIP-

### 2.4 RKIP的表达情况与肺癌临床病理特征的关系

见表 1。RKIP mRNA及蛋白的表达量与患者的性别、年龄、吸烟状态、肿瘤瘤体大小、病理分型均无关（*P* > 0.05）。随着NSCLC的分化越差，RKIP表达量越低，差异有统计学意义（*P* < 0.05）；在pTNM分期中，Ⅰ期+Ⅱ期NSCLC RKIP表达量高于Ⅲ期+Ⅳ期，两组相比有统计学差异（*P* < 0.05）；术后生存期 < 2年者RKIP表达量低于≥2年者，两组相比有统计学差异（*P* < 0.05）；RKIP表达量低的患者更易发生淋巴结转移（*P* < 0.05）。

**1:** 肺癌组织中RKIP的表达与肺癌临床病理特征的关系 Correlation between the expression of RKIP with the clinicopathological characteristics of lung cancer

Variable	*n*	RT-PCR	*t*	*P*	Western blot	*t*	*P*	RKIP+(%)	*χ*^2^	*P*
Gender			0.021	0.984		0.149	0.889		0.312	0.576
Male	48	1.217±0.534			0.612±0.106			19 (39.58)		
Female	35	1.209±0.378			0.598±0.124			16 (45.71)		
Smoking status			0.005	0.996		0.182	0.864		0.690	0.406
Yes	43	1.213±0.326			0.617±0.205			20 (46.51)		
No	40	1.208±0.427			0.587±0.198			15 (37.50)		
Age (yr）			0.006	0.995		0.035	0.974		1.627	0.202
< 50	43	1.219±0.423			0.629±0.212			21 (48.84)		
≥50	40	1.217±0.358			0.623±0.206			14 (35.00)		
Diameter (cm）			0.003	0.998		0.009	0.993		0.017	0.898
< 3	42	1.219±0.479			0.621±0.213			18 (42.86)		
≥3	41	1.218±0.378			0.619±0.304			17 (41.46)		
Histological type			0.017^*^	0.988		0.017^*^	0.987		0.535	0.911
Squamous cell carcinoma	32	1.217±0.372			0.623±0.235			15 (46.88)		
Adenocarcinoma	40	1.212±0.367			0.620±0.204			16 (40.00)		
Adenosquamous cell carcinoma	8	1.213±0.352			0.598±0.305			3 (37.50)		
Large-cell carcinoma	3	1.211±0.377			0.618±0.264			1 (33.33)		
Differentiation			3.270^*^	0.031		2.851^*^	0.046		9.556	0.008
Well	25	1.786±0.224			0.734±0.224			15 (60.00)		
Mediate	23	1.162±0.243			0.318±0.117			12 (52.17)		
Poor	35	1.101±0.004			0.104±0.005			8 (22.86)		
pTNM stage			3.717	0.021		2.869	0.046		4.928	0.026
Ⅰ+Ⅱ	38	1.747±0.213			0.698±0.214			21 (55.26)		
Ⅲ+Ⅳ	45	1.102±0.212			0.306±0.101			14 (31.11)		
Survival time (yr)			3.222	0.032		2.962	0.041		6.119	0.013
≥2	39	1.885±0.376			0.728±0.225			22 (56.41)		
< 2	44	1.148±0.125			0.302±0.107			13 (29.55)		
Lympho invasion			3.093	0.036		3.568	0.023		7.442	0.006
No	40	1.989±0.453			0.737±0.175			23 (57.50)		
Yes	43	1.153±0.118			0.309±0.112			12 (27.91)		
^*^F value.										

## 3 讨论

原发性支气管肺癌恶性度高，其发病率及死亡率呈逐年上升趋势，是一种严重威胁人类健康和生命的疾病。由于肺癌生物学特性十分复杂，其确切的发病机制仍不明确。因此深入研究肺癌的发生机制，寻找更加精确、有效的分子标志物是我们面临的重大难题。

RKIP是一种小分子胞浆蛋白，广泛表达于哺乳动物的心、脑、肝、肺及睾丸等组织，参与神经系统发育、精子产生及细胞凋亡等生理病理过程。研究^[3]^证实RKIP参与细胞内Raf-1/MEK/ERK及NF-κB信号转导途径的调节，通过控制基质金属蛋白酶1/2（MMP-1/2）的表达，从而发挥其促进肿瘤细胞侵润转移的功能。这些信号通路的异常激活还与多种肿瘤的发生发展密切相关，研究发现RKIP表达下调与甲状腺癌^[4]^、肝癌^[5]^、黑色素瘤^[6]^、结直肠癌^[7]^、前列腺癌^[8]^、乳腺癌^[9]^等恶性肿瘤的转移及预后较差有关。

Lee等^[10]^采用PCR方法检测17例肝癌和对应的癌旁组织中RKIP mRNA的表达，结果表明两组差异无统计学意义。本研究PCR结果显示，肺癌组织及癌旁对应肺组织中RKIP mRNA的表达差异有统计学意义，说明在肺癌的发生中，RKIP表达减少可能是在转录水平受到调节，上述研究结果的差异可能与不同的肿瘤组织类型有关。

RKIP具有促进凋亡、抑制肿瘤转移的重要功能，是一种肿瘤转移抑制基因。本研究结果显示，在肺癌组织中无论RKIP在mRNA水平还是在蛋白水平上均明显低于癌旁正常组织；低分化肺癌组织中RKIP的表达水平明显低于高、中分化肺癌组；随着临床分期的增加，RKIP的表达量下降，生存期≥2年者RKIP表达水平高于生存期 < 2年者；有淋巴结转移组RKIP的表达水平明显低于无淋巴结转移组；但与患者的性别、年龄、吸烟、肿瘤的大小及病理类型无关。提示RKIP mRNA及蛋白的表达水平随着肺癌淋巴结转移的发展而呈现下调的趋势，并且RKIP低表达者生存期短。因此，对RKIP mRNA及蛋白表达的检测可作为评估患者淋巴结转移及预后的因素之一，RKIP可作为人类肺癌的转移抑制基因，为肺癌的治疗提供一个新的途径。

总之，RKIP的表达减弱或缺失在肺癌的发生发展以及侵润转移过程中可能起着重要的作用，RKIP参与多条信号通路的调控，这些通路之间的关系如何，RKIP表达减少或缺失的机制是什么，是否与基因缺失、突变、表达产物的失活或基因的甲基化有关，需要进行更深入的研究。通过对肺癌中*RKIP*基因在mRNA水平和蛋白水平表达的研究，有助于了解RKIP的失活机制及与肺癌侵袭、转移的关系，RKIP有望成为肺癌治疗尤其是抑制肺癌侵袭转移的一个有价值的靶点。
